# Dynamic coupling between slow waves and sleep spindles during slow wave sleep in humans is modulated by functional pre-sleep activation

**DOI:** 10.1038/s41598-017-15195-x

**Published:** 2017-11-03

**Authors:** Juliana Yordanova, Roumen Kirov, Rolf Verleger, Vasil Kolev

**Affiliations:** 1 Department of Neurology, University of Lübeck, Lübeck, Germany; 2 Institute of Neurobiology, Bulgarian Academy of Sciences, Sofia, Bulgaria; 3 Institute of Psychology II, University of Lübeck, Lübeck, Germany

## Abstract

Co-existent sleep spindles and slow waves have been viewed as a mechanism for offline information processing. Here we explored if the temporal synchronization between slow waves and spindle activity during slow wave sleep (SWS) in humans was modulated by preceding functional activations during pre-sleep learning. We activated differentially the left and right hemisphere before sleep by using a lateralized variant of serial response time task (SRTT) and verified these inter-hemispheric differences by analysing alpha and beta electroencephalographic (EEG) activities during learning. The stability and timing of coupling between positive and negative phases of slow waves and sleep spindle activity during SWS were quantified. Spindle activity was temporally synchronized with both positive (up-state) and negative (down-state) slow half waves. Synchronization of only the fast spindle activity was laterally asymmetric after learning, corresponding to hemisphere-specific activations before sleep. However, the down state was associated with decoupling, whereas the up-state was associated with increased coupling of fast spindle activity over the pre-activated hemisphere. These observations provide original evidence that (1) the temporal grouping of fast spindles by slow waves is a dynamic property of human SWS modulated by functional pre-sleep activation patterns, and (2) fast spindles synchronized by slow waves are functionally distinct.

## Introduction

Slow wave activity (SWA) and sleep spindles are fundamental electrophysiological signatures of non-rapid eye movement (NREM) sleep in both humans and animals. Cellular recordings in animals have revealed that these two sleep events are not independent during thalamo-cortical interactions^1^. Specifically, sleep spindles in animals may be coupled with the depolarizing up-state of cortical slow oscillations characterized by wake-like cortical activation^1–3^. In humans, sleep spindles also can be grouped by slow oscillations during NREM sleep^4–8^ whereby the grouping by the depolarizing up-state is observed for fast (13–16 Hz) spindles, whereas slow (9–12 Hz) spindles appear coordinated by the emerging hyperpolarizing down-state marked by cortical inactivation^6,7^. The neurodynamic characteristics and possible functions of temporally locked slow-wave and spindle activities have become a focus of increasing interest^9^.

According to previous studies in humans, the entrainment of slow oscillations during slow wave sleep (SWS) by means of transcranial direct current stimulation^10^, transcranial magnetic stimulation^11^ or auditory stimulation^12^ induces a simultaneous increase in slow and fast spindle activities along with an improvement of declarative memory. This has raised the question if combined or isolated modulations of slow waves and sleep spindles affect post-sleep memory^13^. In support to the notion that co-occurring SWA and sleep spindles potentiate sleep-dependent memory consolidation^9,13–15^, a variety of studies have correlated coordinated changes with behavioural improvements after sleep^6,12,16–18^, as well as with general mental ability^19^. However, it remains less well known whether the temporal locking between slow wave and spindle activities vary as a function of preceding brain activations in a use-dependent way^9^.

Notably, previous research has revealed that SWA and sleep spindles are associated with pre-sleep activations. Topographically focused local increases of SWA during NREM sleep emerge after learning at those particular cortical regions that have been most activated during pre-sleep learning^20^, and accordingly, SWA decreases at the areas of daytime inactivation^21^. Likewise, topographic patterns of local spindles are modulated by the material to be processed before sleep^22–26^. Although the general implication of these observations is that both SWA and sleep spindles trace the offline re-activation of functionally relevant areas, the involvement of temporally locked SWA and sleep spindles has not been elucidated. Therefore, the objective of the present study was to explore if the topographic patterns of coupling between co-existing slow-wave and sleep spindle activities depend on pre-sleep functional activations.

**Figure 1:**
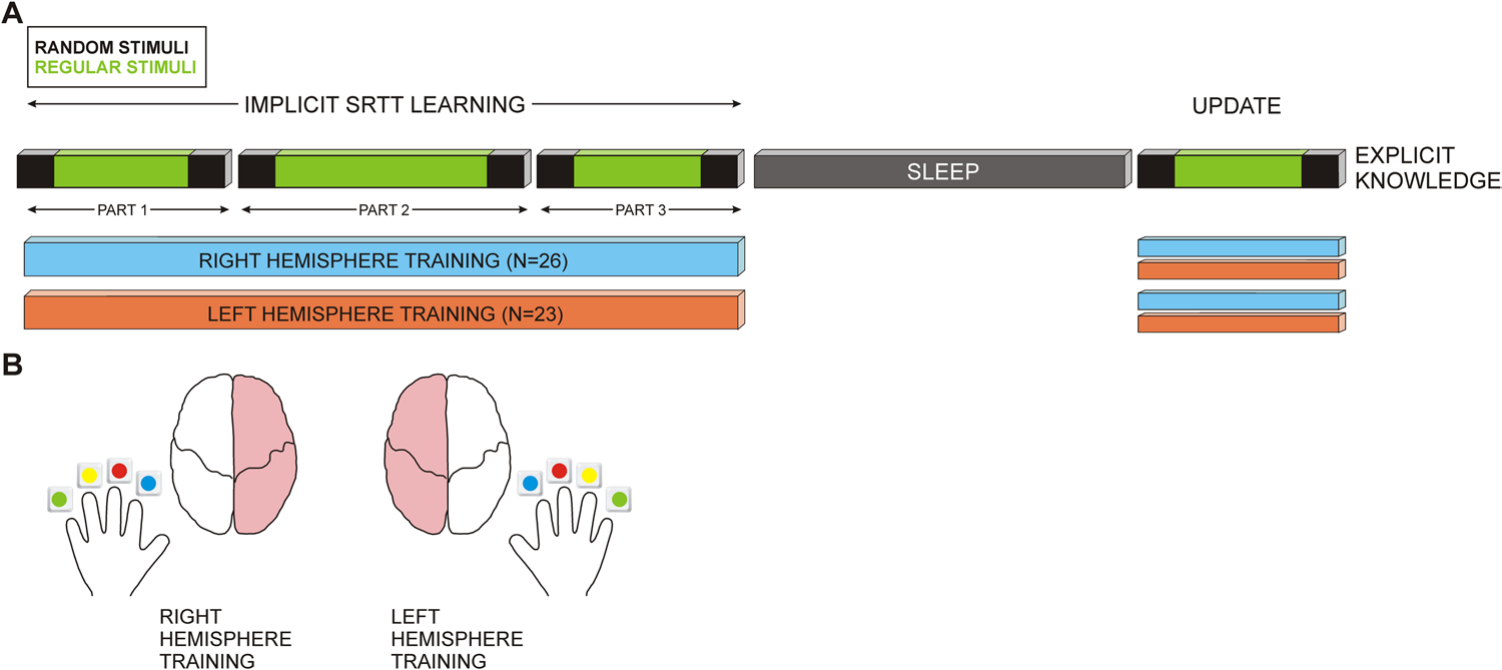
Graphic illustration of the experimental design. (**A**) A serial response time task (SRTT) was performed in blocks containing random or regular stimuli as indicated in black and green, accordingly. Random stimuli appeared before and after regular stimuli (“sandwich” structure) in 3 parts (**PART 1**, **PART 2**, **PART 3**) before sleep (**IMPLICIT SRTT LEARNING**) and in an **UPDATE** session after sleep. Short breaks were introduced between the parts, not between random and regular blocks. Following the balanced design of the experiment, approximately half of the participants performed the task either on the left side (**RIGHT HEMISPHRE TRAINING**) or on the right side (**LEFT HEMISPHERE TRAINING**). Not relevant to the present study, the update session was performed either on the same or on the opposite side. The amount of **EXPLICIT KNOWLEDGE** about the SRTT sequence was measured at the end of experiment. (**B**) Schematic presentation of the stimuli and their correspondence to the required finger to be pressed as a motor response.

To address this question, we manipulated pre-sleep functional activations by engaging differentially the left and right hemispheres before sleep. We used a lateralized variant of a visuo-motor serial response time task (SRTT, see Fig. 1) with a hidden regular sequence^27^. In a learning night, about half of the participants (n = 26) learned the lateralized SRTT before sleep on the left and the other half (n = 23) on the right side, in order to activate predominantly visual and motor cortices of the hemisphere contralateral to the side of learning^28^. Participants were not informed about the presence of the regular sequence. In addition to left or right hemisphere training, we accounted for other factors that might induce inter-hemispheric asymmetry during pre-sleep task performance. Implicit SRTT learning depends on individual strategies^27,29,30^ which may activate differentially the left and the right hemisphere. Specifically, participants who accumulate implicit sequence knowledge during training^29^ may manifest specific activation patterns in the right hemisphere^31^ and participants who subsequently become aware of the hidden sequence may activate more strongly executive control networks and networks in the right hemisphere already during training^27,30,32,33^. With regard to possible strategy-dependent asymmetries, we computed the gain of implicit knowledge about the sequence before sleep and the amount of explicit knowledge about the sequence after sleep and used these measures as control variables in all subsequent analyses of asymmetry.

We verified the pre-sleep lateralized activations by recording electroencephalographic (EEG) signals at multiple (26) electrodes during training. Previous EEG, magnetoencephalographic and corticographic recordings have documented that sensorimotor ‘mu’ rhythms in the alpha (8–13 Hz) and beta (14–24 Hz) frequency ranges decrease (desynchronize) at central and parietal scalp regions as a sign of cortical excitation during preparation and execution of voluntary movements^34–40^. Also, alpha desynchronization at occipito-parietal regions reflects the functional involvement of visual areas^41–44^. Hence, we validated pre-sleep inter-hemispheric differences by the topographic patterns of alpha and beta EEG desynchronization.

The effect of pre-sleep activation on the temporal links between co-existing slow-wave and sleep spindle activities was assessed by comparing inter-hemispheric patterns of coupling in a learning and a non-learning night. We recorded multichannel EEG signals during slow wave sleep when the expression and identification of SWA was most reliable^45^. The coupling between the phase of slow waves and sleep spindle activity^18^ was measured by applying an algorithm capable of quantifying the stability and timing of coupling independently of signal magnitude^46,47^. It was expected that if SW-spindle coalescence was affected by the differential functional activations of the two hemispheres before sleep, asymmetries would be observed between the coupling of the trained and untrained hemispheres.

Behavioural and EEG data from this task have been analysed before^26,27,29,31^ to explore the online and offline mechanisms of awareness of implicitly learned regularities, but the coupling of spindles to SWA has never been taken into account. What is new in the present study are analyses of whether and how spindle activity is coupled to the slow oscillations, and whether these couplings reflect inter-hemispheric differences in preceding functional activations. Accordingly, correlations with offline memory consolidation as reflected by post-sleep SRTT performance were not targeted.

## Results

### SRTT performance

The performance of left- and right-side groups (Fig. 1) was evaluated for SRTT training before sleep. To control for the statistical validity of functional asymmetries induced by the side of the trained hemisphere, individual gain of implicit knowledge (ImK) before sleep and amount of explicit knowledge (ExK) after sleep were included as covariates in the analyses. Individual ImK was computed as the normalized difference between RTs in the random and the preceding regular blocks in the last learning session. The difference would reflect the extent to which regularity violation would induce performance slowing, which would only occur upon implicit sequence acquirement^29^. As applied previously by Yordanova and coworkers^29^, individual amount of ExK after sleep was scored from 1 to 5 depending on the number of items in a sequence that could be correctly re-constructed after sleep (for details, see Methods). Reaction time (RT) was measured in the random and regular blocks in all parts of the learning session to test for the presence of individual differences in sensorimotor processing and learning.

As expected for our sample with right-handers, reactions were faster for right- than left hand responses (F(1/45) = 6.9, p = 0.01). However, online sensorimotor learning (RT speeding in random blocks from the beginning to the end of pre-sleep SRTT learning) did not differ between the left- and right-side learning groups (F(1/45) = 0.04, p > 0.8). Nor did the groups differ in online implicit sequence learning. This was reflected by non-significant between-group differences for RT speeding in regular blocks from the beginning to the end of the pre-sleep learning session (F(1/45) = 0.005, p > 0.9). Accordingly, the amount of implicit knowledge about the sequence gained before sleep reflected by ImK did not differentiate left- and right-side performers (F(1/46) = 2.0, p > 0.15).

### Dynamic characteristics of the coupling between slow waves and spindle activity

In the present study, we analysed how spindle activity was coupled by the distinct cortical functional states fluctuating in the course of the continuous slow waves (SWs) during SWS. To isolate reliably down states indexed by negative phases and up states indexed by positive phases^1^, negative and positive extremes of ongoing SWs were identified and averaged separately. As a result, averaged half waves, SOmin and SOmax, were computed (see details in Methods) and used for further analysis. It is to be emphasized that the waveforms of the half waves extracted by averaging were not considered as distinct oscillatory patterns, but were only used to evaluate spindle grouping by cortical states of activation and inactivation.

To characterize the temporal dynamics of spindle activity around SWmin and SWmax, power of envelopes of slow (9–12 Hz) and fast (13–16 Hz) spindle activity were calculated. Congruency (synchronization) of envelope maxima around SWmin and SWmax was computed^46,47^ by building a histogram of synchronized maxima (see Methods) and identifying histogram peaks. Histogram peaks and their time positions were measured at each electrode to assess the stability and timing of coupling between SWs and spindle activity.

**Figure 2:**
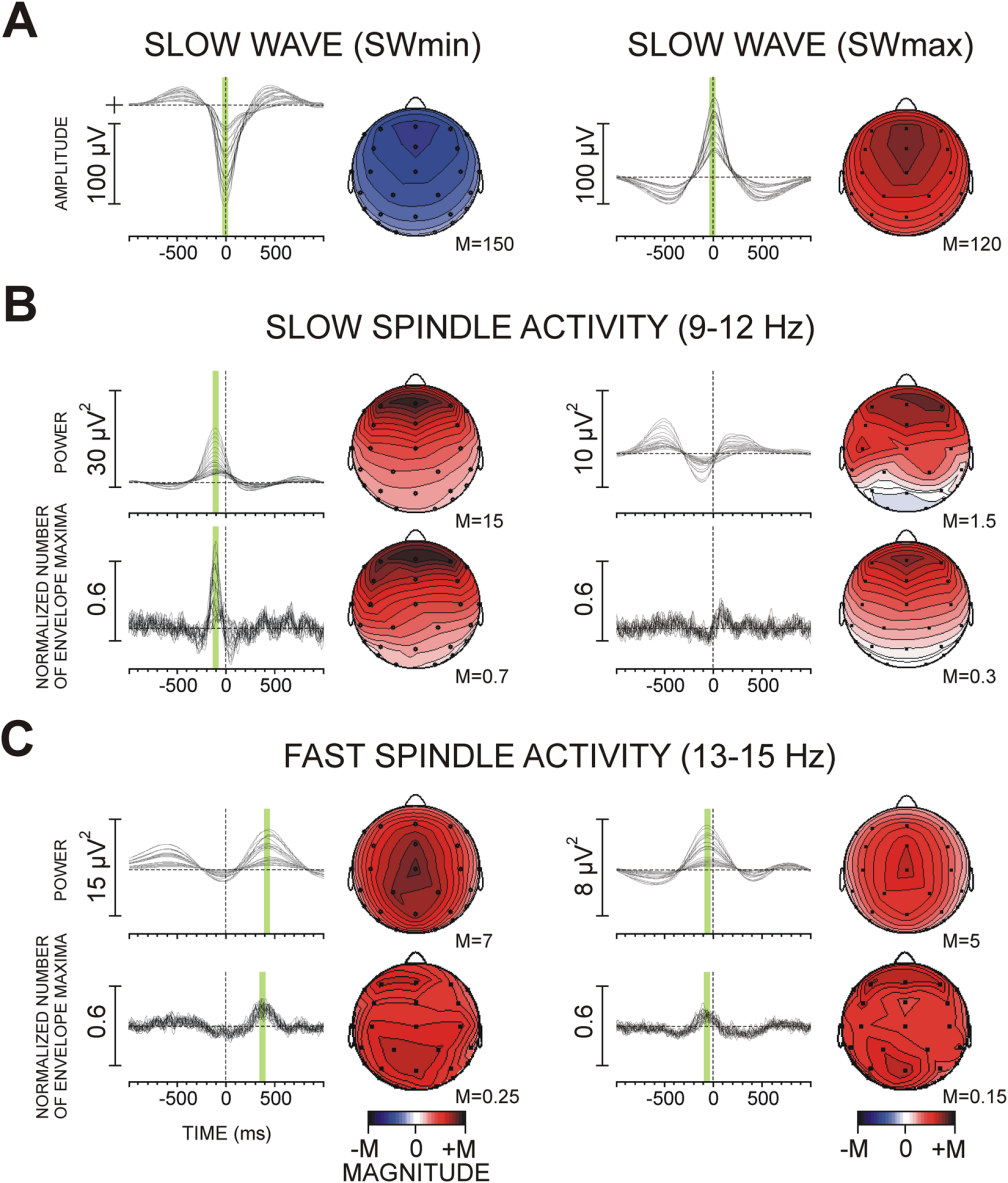
Slow (9–12 Hz) and fast (13–15 Hz) spindle activity coupled with slow half waves (**SWmin** and **SWmax**) during SWS in the non-learning night: group averages of single electrodes superimposed for slow waves, envelope power, and synchronization of envelope maxima (histograms). (**A**) Slow half waves, (**B**) Slow spindle activity, (**C**) Fast spindle activity. The green bars indicate identified maxima used for analysis. Topographic maps of peaks are presented next to each grand average, with magnitude values min-max normalized. Note the different topographies for slow and fast spindle activities.

Figure 2 demonstrates slow (9–12 Hz) and fast (13–16 Hz) spindle activity coupled with SWs during SWS. Group averages of envelope power and synchronization of envelope maxima (histograms) are presented for SWmin (down-state) and SWmax (up-state). Slow spindle activity was enhanced relative to baseline within 300 ms before SOmin (Fig. 2B). This power enhancement was accompanied by a peak in the histogram indicating a maximum in the temporal coupling of slow spindle activity at 122 ms (SE = ± 4.4 ms) before SWmin at Fz (from −125 to −90 ms at different electrodes). There was no reliable power enhancement of slow spindle activity around SWmax (Fig. 2B, right panel).

A different dynamic pattern was found for fast spindle activity. Consistent with the reports of Mölle and coworkers^6^ and Andrillon and coworkers^7^, fast spindle activity was depressed for about 0.5 s around SWmin, but it was enhanced before and after SWmin (Fig. 2C). However, as evidenced by the histogram peak, fast spindle envelopes were temporally coupled only after but not before SWmin (at 363 ms after SWmin at Fz, SE = ± 9.2 ms; from 350 to 415 ms at different electrodes). Also, only fast spindle activity was coupled in relation with the up-state (SWmax) as revealed by a histogram maximum around 100 ms before SWmax. The coupling of fast spindle activity before SWmax was significantly weaker than the coupling of fast spindle activity after SWmin (F(1/45) = 5.8, p = 0.02) – Fig. 2C.

These SW-related spindle activities displayed topographic distribution patterns characteristic for slow and fast spindles (Fig. 2, see also Supplementary Results). Also, the mean duration of averaged spindle activity was between 0.4 s and 0.8 s, thus satisfying the timing criteria for sleep spindle identification (e.g.,^48^).

### Functional asymmetry during pre-sleep SRTT learning

To assess the functional activation during pre-sleep SRRT learning, event-related synchronization/desynchronization (ERS/ERD) was computed for regular and random blocks in the beginning and in the end of training. Low frequency alpha range (α_1_, 8–10 Hz) was analysed to reflect global cortical arousal states and high-frequency alpha (α_2_, 9–12 Hz) and beta (β, 15–25 Hz) ranges were analysed to reflect the activation of task-specific neural circuits with circumscribed representation^34,49,50^. ERS/ERD was measured within 200–600 ms after stimulus representing an epoch relevant for sensorimotor and motor processing^51^.

To verify the asymmetric activation of the two hemispheres during SRTT practice on the left or on the right side, ERS/ERD was analysed at bi-lateral sensory, motor and associative regions relevant for SRTT processing. The set of electrodes used for topographic analysis included F3/F4, FC3/FC4, C3/C4, CP5/CP6, T7/T8, P3/P4, PO7/PO8, and O1/O2. These electrodes formed two topography within-subjects factors in a repeated-measures analysis of variance, Region (with eight levels: frontal (F3/4), pre-motor (FC3/4), motor (C3/4), sensorimotor (CP5/6), temporal (T7/8), parietal (P3/4), parieto-occipital (PO7/8) and occipital (O1/2)) and Laterality (with two levels: left-hemisphere electrodes F3, FC3, C3, CP5, T7, P3, PO7, O1 vs. right-hemisphere electrodes F4, FC4, C4, CP6, T8, P4, PO8, O2). A between-subjects factor was the Trained hemisphere (left hemisphere for the group performing the task on the right side vs. right hemisphere for the group performing the task on left side, see Fig. 1). Again, to control for the statistical validity of functional asymmetries induced by the side of the trained hemisphere, individual gain of implicit knowledge (ImK) before sleep and amount of explicit knowledge (ExK) after sleep were included as covariates in the analysis. Thus, the final statistical design was a Trained hemisphere x Region x Laterality ANCOVA with two covariates, ImK and ExK. Greenhouse-Geisser estimate of sphericity was applied to main and interactive effects of Region (with more than 2 levels). Original df and corrected p-values are reported. Whenever significant interactions with topography factors (Region x Laterality) were yielded, the effects of Trained hemisphere or covariates were tested at specific electrodes using MANCOVA.

**Figure 3:**
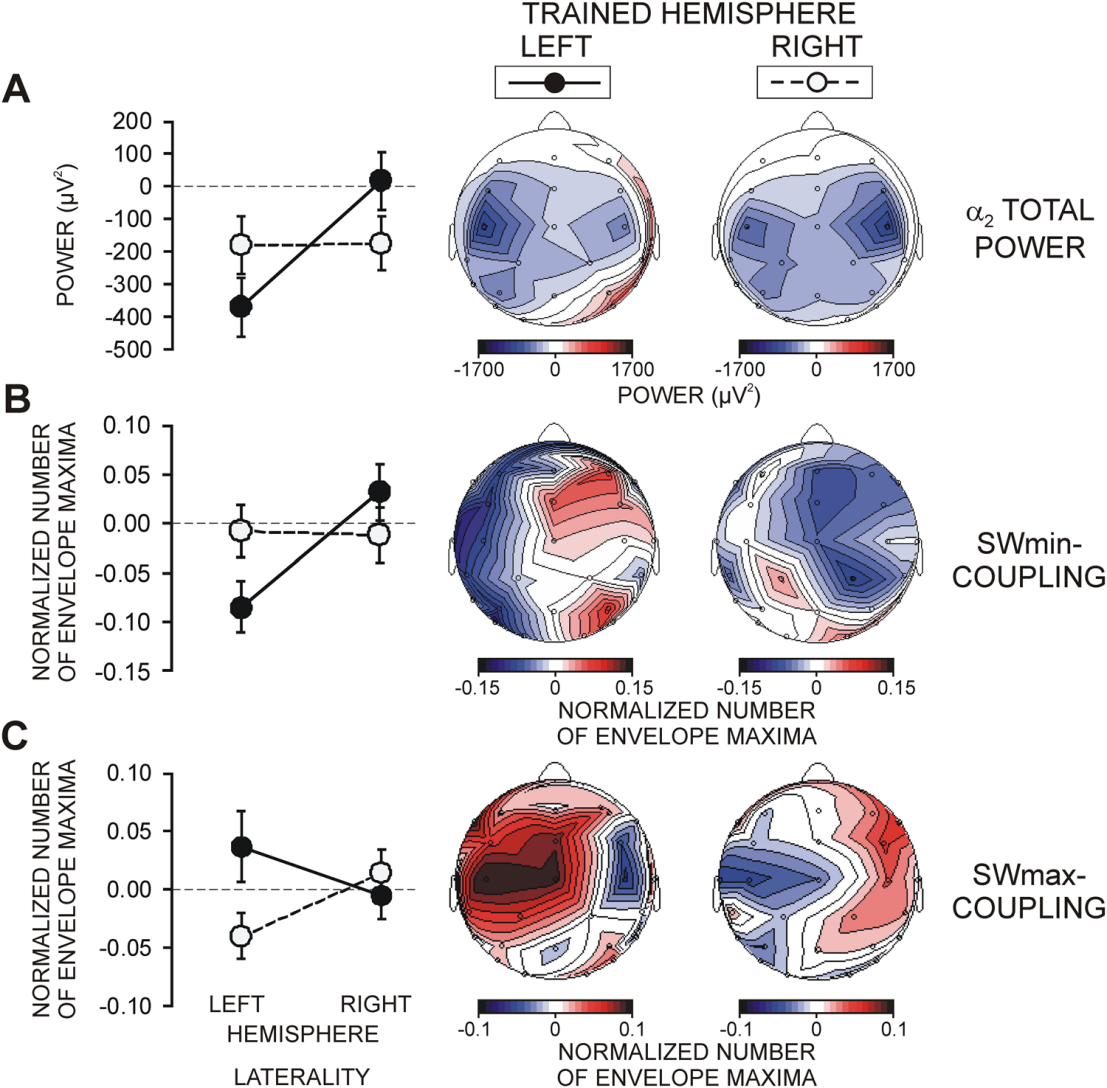
Functional asymmetry between the trained and untrained hemispheres. (**A**) Event-related desynchronization of α_2_ power during task performance (for details, see text). (**B**) Coupling between SWmin and fast spindle activity. (**C**) Coupling between SWmax and fast spindle activity. Difference values in coupling between the learning and non-learning nights are presented. The left panel depicts group mean and SD values for the left and the right hemispheres (**LATERALITY: LEFT**, **RIGHT HEMISPHERE**) from electrodes used for analyses. Maps present the lateral asymmetry for each **TRAINED HEMISPHERE** (**LEFT**, **RIGHT**).

For each frequency band, α_1_, α_2_, and β, functional activation indexed by ERD was revealed during pre-sleep sensorimotor SRTT processing. As depicted in Fig. 3A, activation was overall larger at the left than the right hemisphere as reflected by lateral asymmetry for α_2_ ERD (Laterality, F(1/45) = 5.6, p = 0.02). Predominant activations over parieto-occipital and motor cortical regions were verified by the effects of Region on ERD for each band (F(7/315) > 2.8, p < 0.05). Critically, a pronounced functional asymmetry was observed due to a stronger activation of the trained as compared with the untrained hemisphere indexed by significant Trained hemisphere x Laterality interactions for α_2_ (F(1/45) = 9.3, p = 0.004) and β bands (F(1/45) = 11.05, p = 0.002), and a marginally significant interaction for α_1_ (F(1/45) = 3.3, p = 0.07).

**Figure 4:**
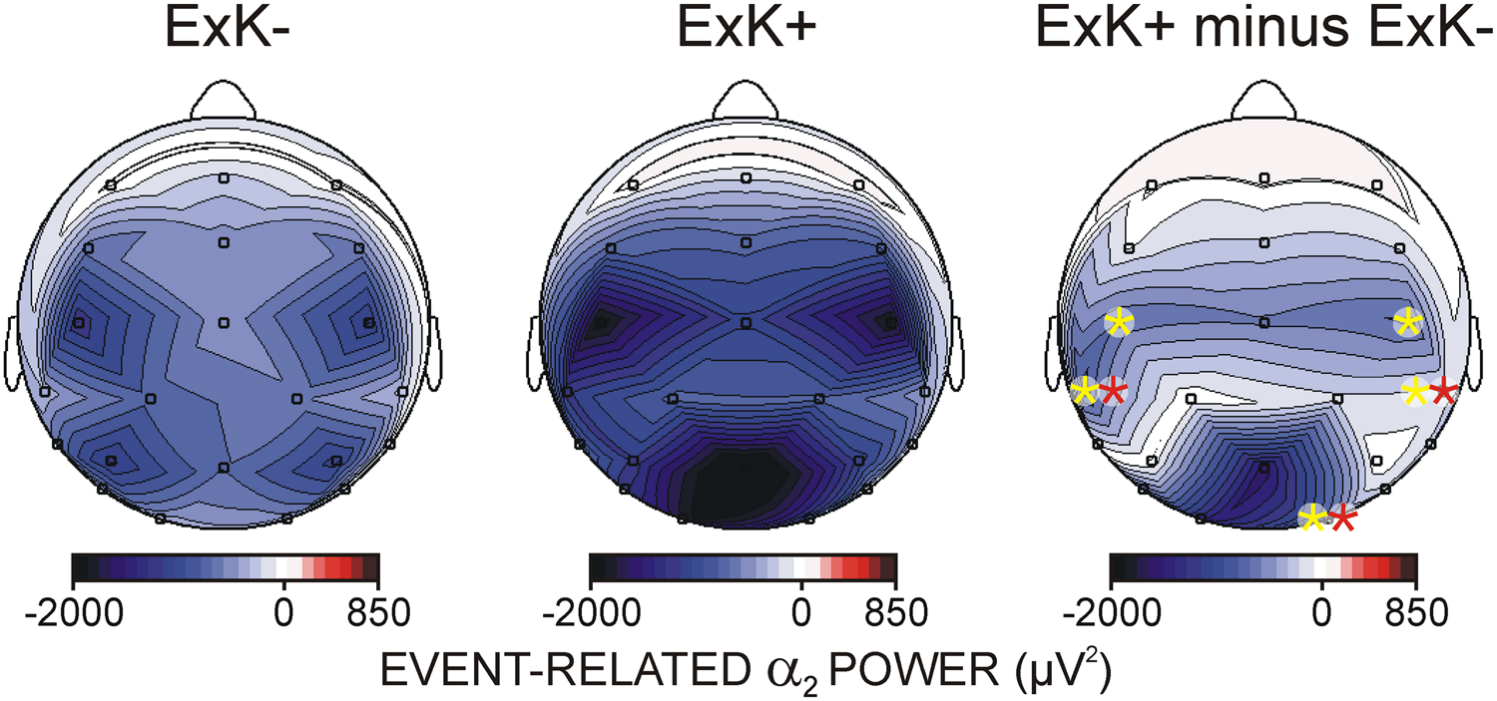
Effects of explicit sequence knowledge after sleep (ExK) on functional activation before sleep. For visualization, participants with low ExK scores (1,2) and low amount of awareness about the sequence after sleep are grouped (**ExK−**) to be contrasted with participants with higher ExK scores (3,4,5) and high amount of awareness about the sequence after sleep (**ExK+**). Maps present event-related desynchronization of α_2_ power during task performance in ExK−, ExK+ participants, and the difference between the two groups. Yellow asterisks indicate single electrodes where ExK was a significant independent covariate in MANCOVA, indicating a stronger activation as a function of the amount of post-sleep ExK. Red asterisks indicate single electrodes where ExK was yielded as an independent predictor of increased SWmax-fast spindle activity coupling after learning.

Figure 4 demonstrates that pre-sleep functional activations predicted the ability of individuals to express awareness of the regularity after sleep. ERD in each frequency band revealed that the amount of ExK was associated with significantly greater functional activation at motor and parieto-occipital regions (Region x ExK covariate for α_1_ F(7/315) = 4.7, p = 0.005; for α_2_ F/315) = 5.0, p = 0.002; for β F(7/315) = 10.5, p < 0.001). These effects were most pronounced at C3, CP5, C4, CP6 and O2 electrodes (Laterality x Region x ExK covariate for α_1_ F(7/315) = 4.2, p = 0.006; for α_2_ F(7/315) = 5.02, p = 0.002; for β F(7/315) = 9.5, p = 0.001), where ExK was a significant independent predictor of ERD (F(1/45) > 4.7, p < 0.05).

### Functional asymmetry of SW-coupled spindle activity

The distribution of sleep stages and sleep efficiency did not differ between participants with left or right-side learning in either the non-learning or in the learning night (see also Supplementary Results and Table S1).

The effect of pre-sleep learning on SW-coupled spindle activity was evaluated using the difference values between learning and non-learning nights. To achieve normalization, the difference was computed for each analysed parameter (*diff*) as the rate of change according to the equation:

1 $$diff=\frac{(Learning\,Night)\ast 100}{Nonlearning\,Night}-100[{\rm{ \% }}].$$

As for ERS/ERD, an ANCOVA was applied with factors Trained hemisphere x Region x Laterality and covariates ImK and ExK. The topography factor Region included motor and parieto-occipital regions that manifested greatest functional activation during SRTT learning indexed by ERD (Fig. 3A). Accordingly, bi-lateral electrodes at six regions (pre-central FC3/4, central C3/C4, post-central CP5/6, parietal P3/4, parieto-occipital PO7/8, and occipital O1/2) were used to form the levels of the factor Region and the levels of the factor Laterality (left hemisphere vs. right hemisphere). As indicated in Fig. 2 (green bars), SWmin-coupled slow spindle activity, SWmin-coupled fast spindle activity, and SOmax-coupled fast spindle activity were analysed. The peaks of SW-related spindle envelopes and peak histogram values were used. It was expected that if the greater activation of the trained hemisphere affected the coupling between SWs and spindle activity, significant Trained hemisphere x Laterality interactions would be yielded.

No significant effects were observed for envelope peaks of slow or fast spindle activity. Nor was the coupling of slow spindle activity by SWs affected. In contrast, inter-hemispheric asymmetry was found for the coupling between SWs and fast spindle activity, as described below.

#### Functional asymmetry of SWmin-fast spindle coupling

Fig. 3B demonstrates that pre-sleep SRTT learning produced laterally asymmetric coupling of fast spindle activity with SOmin due to an overall decrease of coupling over the left hemisphere (Laterality, F(1/45) = 11.9, p = 0.001). Importantly, the SOmin-fast spindle coupling differed between the two hemispheres depending on which hemisphere was trained before sleep (Trained hemisphere x Laterality, F(1/45) = 6.4, p = 0.01). Training the left hemisphere in the right-side group was associated with a significant reduction of coupling over the same (left) as compared to the untrained (right) hemisphere (Laterality, F(1/20) = 17.6, p < 0.0001). Likewise, training the right hemisphere in the left-side group reduced coupling over the trained right hemisphere, but non-significantly as compared to the untrained left hemisphere (Laterality, F(1/23) = 1.5, p > 0.1). Accordingly, a training-related decoupling produced a significant difference between the groups with opposite sides of training over the left hemisphere (Trained hemisphere, F(1/45) = 4.1, p = 0.05) and a trend over the right hemisphere (F(1/45) = 2.8, p = 0.1). ImK and ExK covariates did not yield significant effects.

#### Functional asymmetry of SWmax-fast spindle coupling

Fig. 3C demonstrates that SWmax-fast spindle coupling also was laterally asymmetric depending on which hemisphere was trained before sleep (Trained hemisphere x Laterality, F(1/45) = 4.6, p = 0.03). However, the greater pre-sleep activation of the trained hemisphere affected coupling in opposite directions as compared to SWmin by producing an increase over the trained hemisphere. Again, this effect was pronounced and significant over the left hemisphere, where coupling increased when this hemisphere was trained before sleep and decreased when this hemisphere was not trained before sleep (Trained hemisphere, F(1/45) = 5.4, p = 0.02). Comparing the trained and untrained hemispheres in each of the groups with left- or right-hemisphere training did not yield reliable asymmetries.

Notably, as Fig. 4 shows, the amount of post-sleep ExK about the sequence predicted the increase in SOmax-fast spindle coupling at specific regions, independently of which hemisphere was trained before sleep (Region x Laterality x ExK, F(5/225) = 4.4, p = 0.001). Using MANCOVA, the independent ExK effect was tested at single electrodes. A significant continuous prediction of ExK was found at CP5, CP6, and O2 electrodes (F(1/45) > 4.04, p < 0.05), i.e., electrodes where the amount of ExK was associated with increased activation during training.

### Control analyses

#### Slow waves

To test if asymmetric coupling was guided by the magnitude of slow waves, peak amplitudes if averaged negative and positive half slow waves were analysed. Normalized difference values between the learning and non-learning nights were computed for peak amplitudes of SOmin and SOmax and were subjected to a Trained hemisphere x Region x Laterality ANCOVA with ImK and ExK covariates. No inter-hemispheric asymmetries relevant to the Trained hemisphere were found for either SOmin or SOmax amplitudes (Trained hemisphere x topography factors, F < 1.8, p > 0.2).

#### Correlational analyses

The association between the post-learning SWmin-fast spindle decoupling and the post-learning increase in SWmax-fast spindle coupling was tested using Pearson correlation coefficients. At none of the electrodes was there a correlation between the post-learning changes in the coupling related to SWmin and SWmax (p > 0.3). In line with the absence of significant learning-dependent changes in the amplitudes of SWmin and SWmax, these changes did not correlate with the coupling of the related fast spindle activity, indicating that the magnitude of SWs was not the critical determinant of the capacity of SWs to group fast spindle activity. Also, no significant correlations existed at specific electrodes between alpha and beta ERD values during SRTT learning and coupling between slow waves and fast spindle activity during sleep.

## Discussion

Recent sleep studies imply that major neuroelectric events during NREM sleep, sleep spindles and slow oscillations, support concurrently behavioural improvement after sleep (e.g.,^10,16,17^). Co-existent sleep spindles and slow oscillations may therefore represent a functional mechanism for offline information processing^9^. In the present study, we explored if the temporal synchronization between slow waves and spindle activity during SWS was modulated by pre-sleep learning and if specific cortical pre-activations played a role for such variations. For that aim we activated differentially the left and right hemisphere before sleep by using a lateralized variant of SRTT and verified these inter-hemispheric differences by analysing alpha and beta EEG activities during learning^34,35^. Major results demonstrate that the temporal synchronization between the emerging cortical up state of slow waves and fast spindle activity during SWS was laterally asymmetric, corresponding to hemisphere-specific activations before sleep. These observations provide original evidence that the temporal grouping of fast spindles by slow waves is a dynamic property of human SWS modulated by functional pre-sleep activation patterns.

The approach we used to quantify coupling did not apply dichotomous criteria for the presence or absence of spindles^48,52,53^. Rather, spontaneous modulations of spindle power were evaluated to provide an objective measure of the timing of spindle activity around slow waves. This new approach revealed that spindle-activity maxima consistently emerged in fixed temporal windows around SWs, thus verifying the presence of temporal coupling. Analysis of dynamic characteristics demonstrated that slow spindle activity was temporally locked at around 120 ms before SWmin, the waning down-state of SW, whereas fast spindle activity was temporally locked mainly with respect to SWmax, the up-state of SW in two distinct windows, one emerging late (around 300–400 ms) after the SW down-state, and the other emerging shortly before (around 100 ms) the SW up-state. These observations are fully consistent with previous research where discrete spindles were identified around the SWmax using standard EEG criteria^5,6^, EEG/MEG criteria^54^ and electro-corticographic recordings^7^, pointing to the primary contribution of classical spindle events to the clusters of coupled spindle activity captured by our approach. Further, the characteristics of averaged spindle activity computed here satisfied spindle identification criteria in terms of frequency, topography, duration and magnitude^52^. The distinct involvement of slow and fast spindle activity in the temporal evolution of slow waves supports the notion that slow and fast spindles represent different classes of sleep events^53^ with possibly separate sources of generation^6^.

The inclusion of multiple regions in the present analysis demonstrated that the coupling of fast spindle activity with the SW down state was destabilized at the trained hemisphere that had a greater functional pre-activation before sleep. In contrast, the coupling between SW up-state and fast spindle activity increased after learning, again following the laterally asymmetric functional involvement of the two hemispheres before sleep. These results provide original evidence that the temporal link between SW and fast spindle activity in both temporal frames is a dynamic feature modulated by task-specific cortical activations before sleep. They corroborate the understanding that sleep spindle activity is modulated by cortical functional states^1^ and strongly emphasize the role of preceding cortical activations during wake in this process.

The opposite inter-hemispheric reactivity of the coupling between SW and fast spindle activity in the two temporal frames (decoupling and increased coupling) (1) may indicate that the two clusters represent different properties, or (2) may reflect a re-distribution of a unitary spindle cluster. Previously, Ruch and coworkers^17^ have found that spindles in stage 2 of sleep tend to occur more frequently during up-states than down-states following learning, suggesting a redistribution of a unitary spindle phenomenon from down- to up-states. Within the redistribution notion, SWmin decoupling in our study would correlate with increased SWmax coupling after pre-sleep learning. However, we found no correlation between the learning-induced modulations of coupling in the two temporal frames, suggesting that the variations of temporal stability might not reflect a unitary phenomenon and might have different origins. These two temporal windows of fast spindle grouping in the course of the emerging cortical up-state have been first described by Mölle and coworkers^5^. Consistent with their report, the post-SWmin coupling measured here was significantly stronger than the coupling roughly coinciding with the SWmax up-state. Mölle and coworkers^5^ have suggested that the spindle activity after slow negative half waves reflects post-inhibitory rebound of spike bursts in thalamo-cortical neurons^55^, whereas spindle grouping before the positive slow half wave reflects the driving influence of cortical up-state depolarization on the generation of spindle oscillations in thalamo-neocortical feedback loops through increased firing of cortico-thalamic projections^1,2^. Hence, functionally different origins of coupling in the two temporal frames have been proposed. The present results support this notion and provide further evidence for a functional distinction. First, we found no correlation between the learning-induced modulations of coupling in the two temporal frames. Second, SWmin-related decoupling was more expressed over the left hemisphere, tracing the overall stronger activation of the left hemisphere during SRTT learning, possibly in relation to the right handedness in the majority of participants. In contrast, the greater task-related activation of motor and sensory regions of participants with subsequent explicit knowledge about the sequence was traced by increased coupling during the depolarizing up state of cortical oscillations. These observations imply that use-dependent decoupling in the emerging up state may be associated with neuroplasticity^19^, whereas increased coupling during the up state may rather support re-activations of task-dependent executive representations.

What may be the behavioural consequence of such modulations of the temporal coupling? It has been demonstrated that external sensory input to the neocortex has the potential to modify cortical responses during cortical up states^12^. However, external sensory input to the neocortex is blocked during spindle generation^56,57^. Hence, learning-dependent increased coupling of fast spindle activity during the up states may specifically protect functional task-related representations reactivated during states of cortical depolarization. Yet, further investigations are needed to highlight these mechanisms and their role for offline information processing.

One explanation of the corresponding asymmetries is that the SW-fast spindle coupling is guided by homeostatic mechanisms^20,21,58^. However, the lack of precise matching in region-specific patterns between pre-sleep activation and coupling (Fig. 3) confirmed by the lack of correlations at specific electrodes between ERD during learning and coupling during SWS, does not provide a direct support to the role of homeostatic mechanisms, although such comparisons may be compromised by spatial resolution constraints of the EEG signal. Further, a function-based explanation can be suggested. Functional magnetic resonance imaging (fMRI) has previously demonstrated greater activations of the premotor, motor and somatosensory cortex, superior parietal lobule, and the thalamus during SRTT learning as compared to simple sensorimotor learning^33^. The present ERD results are consistent with these fMRI observations emphasizing the involvement of contralateral sensory-motor cortical networks during lateralized SRTT practice. More importantly, previous analyses of resting-state networks have demonstrated that residuals of such (sensorimotor or attentional) activities are preserved after task training^59^. Specifically for implicit visuo-motor SRTT, increased connectivity in a network including pre-central and sensory-motor areas has been preserved after learning, with a subsequent involvement of a thalamo-basal network^60^, indicating long-lasting residual activations in the brain.

Hence, residual functional activities may affect the coupling between SWs and fast spindle activity. Such residuals may interact with the emerging slow wave and alter its magnitude at the cortical level. We showed, however, that the amplitudes of averaged slow half waves were not lateralized or correlated with coupling after learning, rending this option questionable. Moreover, in a previous study^54^, lateralized slow oscillations have not been associated with differences in spindle grouping. As another mechanism, residuals in the thalamo-cortical loops^60^ representing functional memory traces in the thalamo-cortical system may be suggested to alter the thresholds of thalamo-cortical firing generating spindle oscillations.

Although the precise neurophysiologic origins and functional correlates of the coupling between spindle activity and slow cortical waves remain to be established, the results of the present study provide evidence that these temporal associations are a dynamic region-specific property modulated by functional pre-activations before sleep.

## Materials and Methods

### Participants

This study was approved by the Ethics Committee of the University of Lübeck, Germany. All subjects provided written informed consent for participation in the study and all methods were performed in accordance with the relevant guidelines and regulations. Fifty-three students at the University of Lübeck (mean 23.4 ± 2.16 years, 28 female) participated. All had normal or corrected to normal vision and normal colour vision according to self-reports. Due to technical reasons 4 subjects were excluded from analyses. All participants were right-handed as evaluated by the Edinburgh Handedness Inventory^61^, without any history of neurologic, psychiatric or sleep disturbances. They were asked to abstain from caffeine six hours before the experiment. The study was conducted in an EEG laboratory of the Department of Neurology, University of Lübeck. Participants received monetary compensation of 60 €.

### Sleep Experiment

Participants practiced a serial response time task in an evening session, and performed an update session in the morning after sleep. They spent an adaptation (non-learning) night in the laboratory with a polysomnographic (PSG) recording. After 7–29 days (median 11 days) they spent the learning night which was preceded by the learning session and followed by the update session (Fig. 1, Supplementary Fig. S1A). For the learning night, participants reported to the laboratory at ∼21:00 h. After placement of electrodes for EEG/PSG recording, they performed three blocks of practice (PART 1, 2, 3 in Fig. 1) and went to bed at ∼23:00 h. After 8 hours in bed, participants were awakened at ∼07:00 h, only from light sleep stages 1 or 2 to avoid cognitive disturbances that can occur after awakenings from SWS or REM sleep. Finally, they performed the update session with EEG recording starting at ∼07:30 h. Subjective levels of sleepiness, activation, boredom, concentration, and motivation were assessed on five-point scales immediately before and after each session of practice (learning) and update.

### Serial reaction time task

#### Stimulation material

The SRTT used in the study was a modification of the standard version introduced by Nissen & Bullemer^62^ and Willingham and coworkers^63^. The task was programmed by means of the Presentation Software version 14.5 (Neurobehavioral Systems, Inc., Albany, USA) and stimuli were presented on a 17″ computer monitor. Participants were instructed to maintain their gaze during the whole experiment to the middle of the monitor. Detailed description of experimental parameters is presented in Supplementary Methods. To control for eyes fixation to screen centre, an eye-tracker was used (Eye-Tracker 600 Series, Eyegaze Edge, LC Technologies, Inc., Fairfax, USA). If fixation deviated from the screen centre by more than 2.6 cm at trial onset (visual angle larger than 1.3°), a large red exclamation mark appeared for 2 s in the middle of the screen attracting gaze back to the centre. Then the trial was restarted.

#### Lateralized task

Throughout any session, responses were given with the same hand, ipsilateral to the constant side of the colour stimuli. Participants were instructed to press the respective button on a response pad as quickly and accurately as possible. The positions of the four buttons corresponded to the position of the fingers of a relaxed freely placed hand (Supplementary Fig. S1B). Approximately half of participants (n = 26) trained the task on the left side, and the other half (n = 23) – on the right side (Fig. 1).

#### Experimental design

The experimental design followed the structure used by Cohen and coworkers^64^. As displayed in Supplementary Fig. S1A, the learning session consisted of three parts of 280, 400, and 280 trials, altogether 960 trials, with self-terminated breaks between parts, and the test consisted of one part of 280 trials. One of the four colours (Blue, Red, Yellow, Green) appeared in each trial and had to be responded by pressing the appropriate key. Untold to participants, each part was a “sandwich” where the outer trials (first 50 and last 50, marked in black) followed a predetermined quasi-random series (but immediate repetitions of the same colour did not occur) whereas the inner trials (180, 300, and 180 in the three parts) repeated a fixed sequence of 12 stimuli (15, 25, and 15 times): B-R-Y-B-G-Y-R-B-Y-G-R-G (Supplementary Fig. S1B). After the update session, participants filled in a questionnaire to probe their explicit knowledge related to the hidden task structure and were asked to write on paper any regular sequence they had noted.

#### Performance parameters

Individual gain of pre-sleep implicit sequence knowledge (**ImK**) was computed as the normalized difference between averaged response times (RTs) in the last random block and RT in the preceding regular block in the last (third) part of the pre-sleep learning session^29^. Individual amount of explicit knowledge about the sequence (**ExK**) was scored from 1 to 5: In case of no regularity being detected or no feeling of any pattern in the stimulation, the participant was scored with 1. Those who could recall a single sequence of 3–4 items were scored with 2; those recalling two correct sequences of 3–4 items each were scored with 3; those recalling a correct sequence of more than 8 items were scored with 4, and participants who were able to report the whole sequence of 12 items were scored with 5. Sensorimotor online learning was measured by the normalized difference between RT in the first random block of Part 1 and the first random block of Part 3 of SRTT learning before sleep and by the normalized difference between RT in the first regular block of Part 1 and the last regular block of Part 3 of SRTT.

### EEG recording and analysis during SRTT performance

EEG was recorded during pre-sleep SRTT learning at the same electrodes with the same parameters used for sleep EEG recording. EEG epochs contaminated with EMG or technical artifacts were rejected. Artifact-free and EOG corrected^65^ event-related single EEG epochs of 1000 ms length and 200 ms pre-stimulus baseline were used. The spatial resolution of EEG was improved by applying current source density transform to EEG^66^. ERS/ERD was computed on single-trial basis with reference to activity in a 200 ms period before stimulus in time windows of 200–400 ms and 400–600 ms after stimulus to reflect sensorimotor and motor activations^51^ at electrodes relevant to the visuo-motor SRTT. To reflect the overall amount of functional activation, the first two blocks in the beginning of learning (random and regular) and the last two blocks in the end of learning (regular and random) were used (marked with arrows in Supplementary Fig. S1A). Mean number of trials in each of the four blocks was 44 (SD = 3.7) because the first 50 trials from block 2 and the last 50 trials from block 11 were taken for analysis (Supplementary Fig. S1A).

### Sleep EEG/PSG recording and analysis

During the whole night, EEG was recorded with Ag/AgCl electrodes (Easycap, http://www.easycap.de) from 25 scalp sites according to the International 10/20 system (F7, F3, F4, F8, FC3, FCz, FC4, T7, C3, Cz, C4, T8, CP5, CP1, CP2, CP6, P7, P3, Pz, P4, P8, PO7, PO8, O1, O2) against a reference positioned on Fz, and Fpz serving as ground electrode. Additionally, bioelectrical signals from both sides of the nose were recorded for off-line reference. Horizontal and vertical electrooculogram (EOG) as well as electromyogram (EMG) from left and right m. masseter were also recorded. Data were amplified with cut-off frequencies DC and 250 Hz by a BrainAmp MR plus (Brain Products GmbH, Gilching, Germany) and stored with a sampling rate of 500/s. EEG was offline re-referenced to the mean value of both nose electrodes, thus providing information about Fz electrode. Analyses were performed by means of Brain Vision Analyzer 2.1 (Brain Products GmbH, Germany) and by custom software developed on Matlab R2013b (The MathWorks Inc.).

Sleep stages (1, 2, 3, 4, and REM sleep), awake time, and movement artifacts were identified offline for 30-s epochs according to Rechtschaffen and Kales^45^ by two experienced sleep raters. EEG recorded during SWS was further analysed to quantify slow oscillations and sleep spindles.

### Coupling between slow waves and spindle activity

#### Detection of slow waves

**Figure 5:**
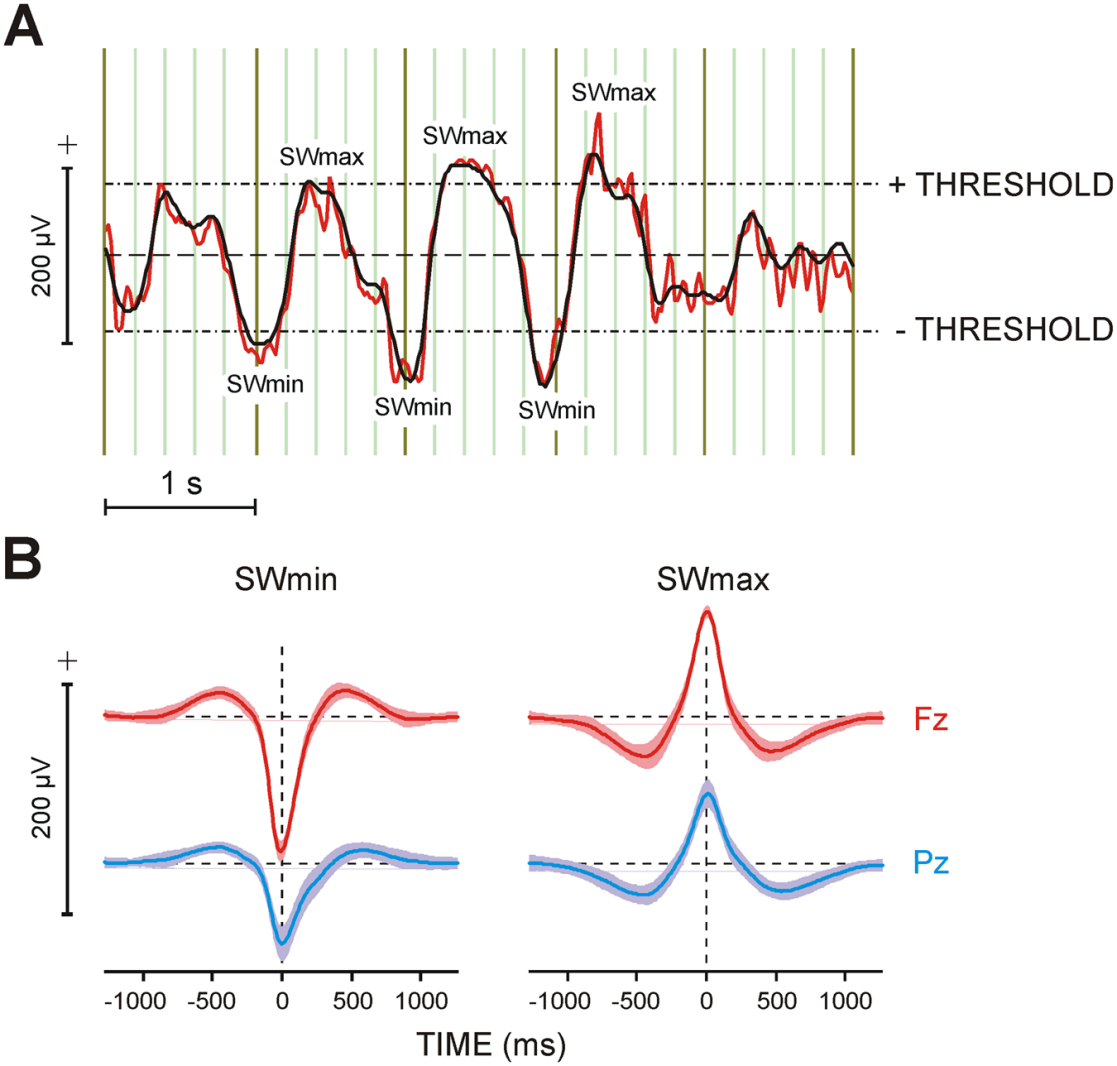
Detection and analysis of slow waves (SWs). (**A**) Procedure for detection of SWs. Typical slow wave EEG recorded during SWS at Fz (red curve) band-pass filtered in the frequency range 0.3–4 Hz (black curve): thresholds and detected SW extrema (**SWmin** and **SWmax**) are presented. (**B**) Grand average slow waves synchronized to their extrema at **Fz** and **Pz** and segmented to **SWmin** and **SWmax** (zero point), accordingly. Shaded areas denote ± 1 standard deviation. The baseline for averaging was mean activity in the time windows 900 to 1200 ms before and after SWmin/SWmax. Dashed lines present zero lines.

Sleep EEG was recorded at 26 electrodes during the learning and non-learning nights. As a first step of the analysis of the coupling between SWA and sleep spindle activity, we performed a detection of slow waves (SWs) in sleep EEG signals. Following Mölle and coworkers^6^, SWs were detected at the frontal electrode Fz. As demonstrated in Fig. 5A, sleep EEG was band-pass filtered within 0.3–4 Hz and the resulting signal was level-triggered with a threshold of ± 80 µV. Peaks exceeding the positive and negative thresholds were marked. SWmin/SWmax markers were thereafter transferred to the original EEG which was segmented for ± 1280 ms around these markers. SWmin/SWmax peaks were aligned to appear at time zero. The first two hundred of the resulting EEG segments were taken for analysis. They fall into the first 40–50 min of SWS, thus covering the SWS stage of the first sleep cycle^6,9^.

These two hundred EEG segments were averaged separately for SWmin and SWmax epochs, with reference to the mean of two baselines of 300 ms duration at 900–1200 ms both before and after the trigger (Fig. 5B). Averages were computed for each subject, electrode, and night (non-learning and learning). Similar to Mölle and coworkers^5^, the algorithm applied here to detect the largest positive and negative deflections in the 0.3–4 Hz filtered signal resulted in averaged half-waves with a length of 0.6–0.8 s (Fig. 5B), corresponding to a frequency of 0.63–0.83 Hz for the oscillation underlying the averaged half-waves. Thus, although raw signals of 0.3–4 Hz were used, the frequency content of the averaged half-waves applied for further analysis fits the frequency range of 0.6–1 Hz as used in previous reports to establish the existence of slow oscillatory rhythms^67,68^, as well as the grouping of spindles by slow oscillations in humans^5,6^.

#### SW-related spindle activity

Since spindles are discrete events of rhythmic oscillations from alpha (9–12 Hz) and sigma (13–16 Hz) frequency bands^48,52^, frequency-specific spectral peaks in SW-related sleep EEG activity would verify the presence of spindle activity in SW-related epochs. To provide this verification, individual single EEG epochs of 2560 ms duration were analysed in the frequency domain by applying the fast Fourier Transform. Observation of individual spectra revealed a consistent spectral peak from the sigma (13–16 Hz, mean 13.4 ± 0.53 Hz) range across all participants at all electrodes. A clear spectral peak in the alpha (9–12 Hz, mean 10.6 ± 0.82 Hz) range with frontal distribution was observed in 25.5% of all participants indicating the presence of rhythmic slow spindle activity. Although a spectral alpha peak was not evident in the rest of the subjects, spectral 9–12 Hz power was enhanced at frontal relative to posterior locations, providing a topographic verification of slow spindle activity. No difference existed between the spectral characteristics of SWmin and SWmax-related epochs, or between left- and right-side learning groups.

#### Temporal coupling between spindle activity and slow waves

Following the outcome of frequency analysis, single-trial SW-related EEG epochs were band-pass filtered in the 9–12 Hz and 13–16 Hz frequency bands. To eliminate fast-frequency activity that may appear as a time-locked component in the averaged slow half wave due to SW sharp transitions between down and up states and vice versa, the averaged slow half wave was subtracted from each single sweep before band-pass filtering^69^. Subtraction was performed for individual and electrode-specific averages of SWmin and SWmax in each night.

**Figure 6:**
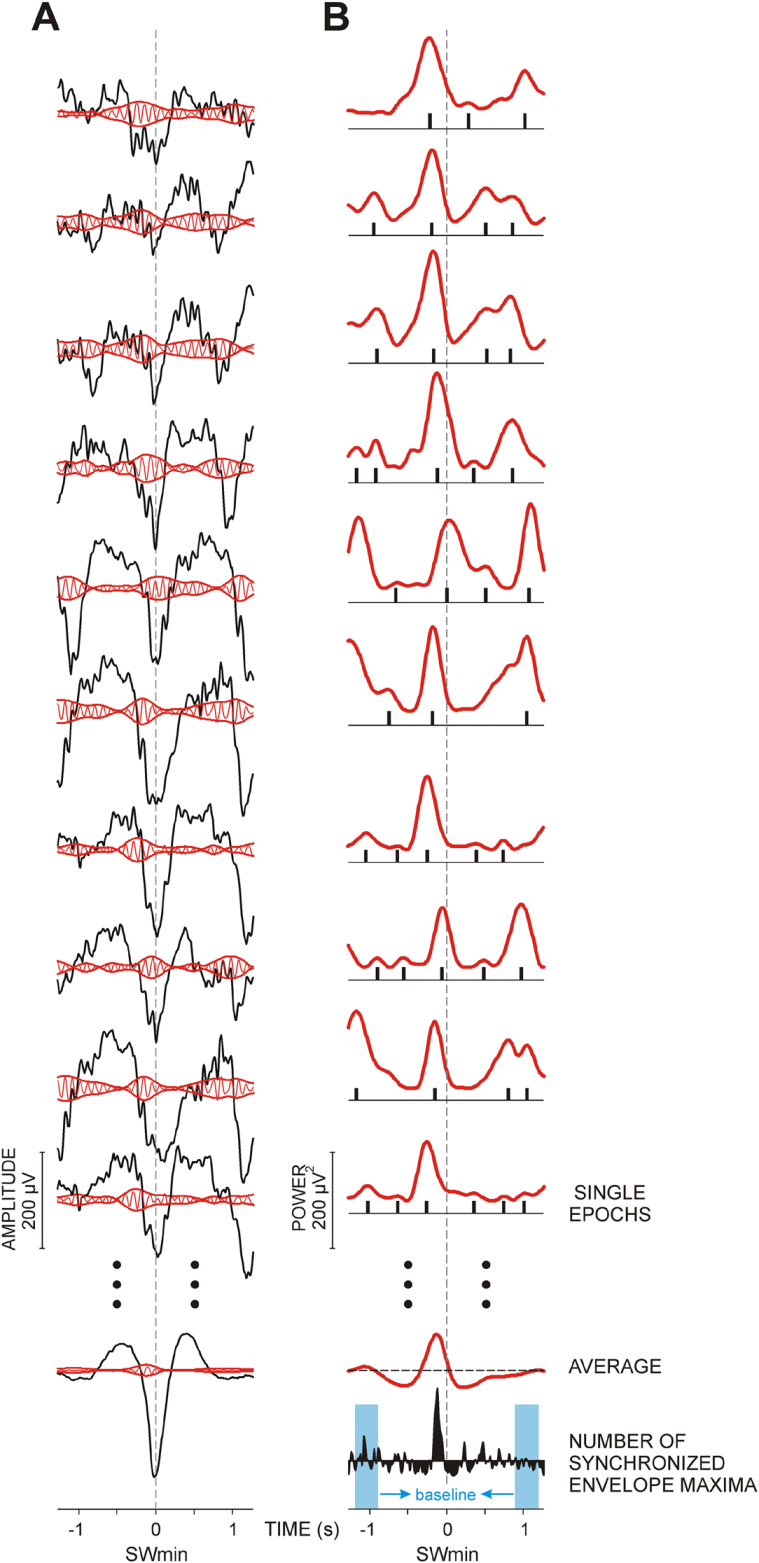
Method and parameters for analysis of the coupling between slow waves and spindle activities. (**A**) Envelope amplitude quantification. Original EEG epochs (black curves) are superimposed with the band-pass filtered EEG (in this example 9–12 Hz, red curves). Envelopes of filtered EEG are presented in red. At the bottom, an average of 200 single epochs is presented. (**B**) Quantification of the phase-synchronization of envelopes. The power of envelopes of the respective filtered epochs calculated by means of the complex demodulation method is presented in red. After averaging, the averaged envelope power is obtained and its maxima are measured and further analysed. To evaluate congruency of envelope maxima between single epochs independently of power, a method sensitive to between-epoch coupling is used. The method determines all local envelope maxima in each single epoch, and produces modified single epochs containing only information about the position of the determined maxima (black bars below the red curves). Each bar for an identified maximum in each single epoch has a value of 1. After averaging of the 200 modified epochs, a histogram of the number of synchronized envelope maxima is obtained (black curve at the bottom). For statistical analyses, the maximal values of histograms are measured for each electrode and condition in each subject. The baseline calculated as mean activity in the time windows 900 to 1200 ms before and after SWmin is presented in blue on the histogram.

To characterize the temporal dynamics of spindle activity, envelopes of subtracted filtered EEG epochs were calculated by means of the method of complex demodulation, which describes the power of the selected frequency range at each point in time^70,71^. Power of single-epoch envelopes was computed and averaged. In this way, the temporal dynamics of alpha/sigma activity modulation is emphasized and any stable temporal grouping of modulated waves was extracted in the average of envelope power as a time-locked component. This procedure is demonstrated in Fig. 6A.

To evaluate congruency of envelope maxima between single epochs independently of power, a method sensitive to between-epoch coupling was used^46,47^. In each single epoch, all maxima of the envelope power signal were identified and marked by + 1 and the remaining data points were set to zero (black bars below the curves on Fig. 6B). By averaging of these modified epochs, a histogram of the occurrence of envelope maxima was obtained. Again, the two intervals of 300 ms duration at 900–1200 ms both before and after the trigger served as baseline (Fig. 6B, bottom). Thereby, randomly occurring maxima were reduced to baseline levels. To ensure data reduction and smoothness, histograms were three-points digitally smoothed and down-sampled to 30 ms dwell time and further normalized by dividing consecutive histogram values by the number of included sweeps. The maximal values of the individual histograms were identified for each electrode and frequency, separately for SWmin and SWmax epochs. Magnitude (normalized number of synchronized envelopes) and latency of the identified maxima in individual histograms were measured and analysed to assess coupling variation and timing.

## Statistics

Statistical evaluation was performed using ANCOVA and Pearson’s correlations (IBM SPSS Statistics, ver. 22.0), with detailed description of designs presented in the related texts.

## Electronic supplementary material

Supplementary Information
