# Supplementary material for: Dynamic coupling between slow waves and sleep spindles during slow wave sleep in humans is modulated by functional pre-sleep activation
Source: Sci Rep. 2017 Nov 3;7:14496. doi: 10.1038/s41598-017-15195-x (PMC5670140; doi:10.1038/s41598-017-15195-x)
Supplement: Supplementary file 1 — Supplementary Information [file 41598_2017_15195_MOESM1_ESM.pdf]

SUPPLEMENTARY INFORMATION

for

Dynamic coupling between slow waves and sleep spindles during slow wave sleep in humans is modulated by functional pre-sleep activation

Juliana Yordanova[1,2], Roumen Kirov[2], Rolf Verleger[1,3], Vasil Kolev[1,2]

[1] Department of Neurology, University of Lübeck, Germany

[2] Institute of Neurobiology, Bulgarian Academy of Sciences, Sofia, Bulgaria

[3] Institute of Psychology II, University of Lübeck, Germany

Supplementary Methods

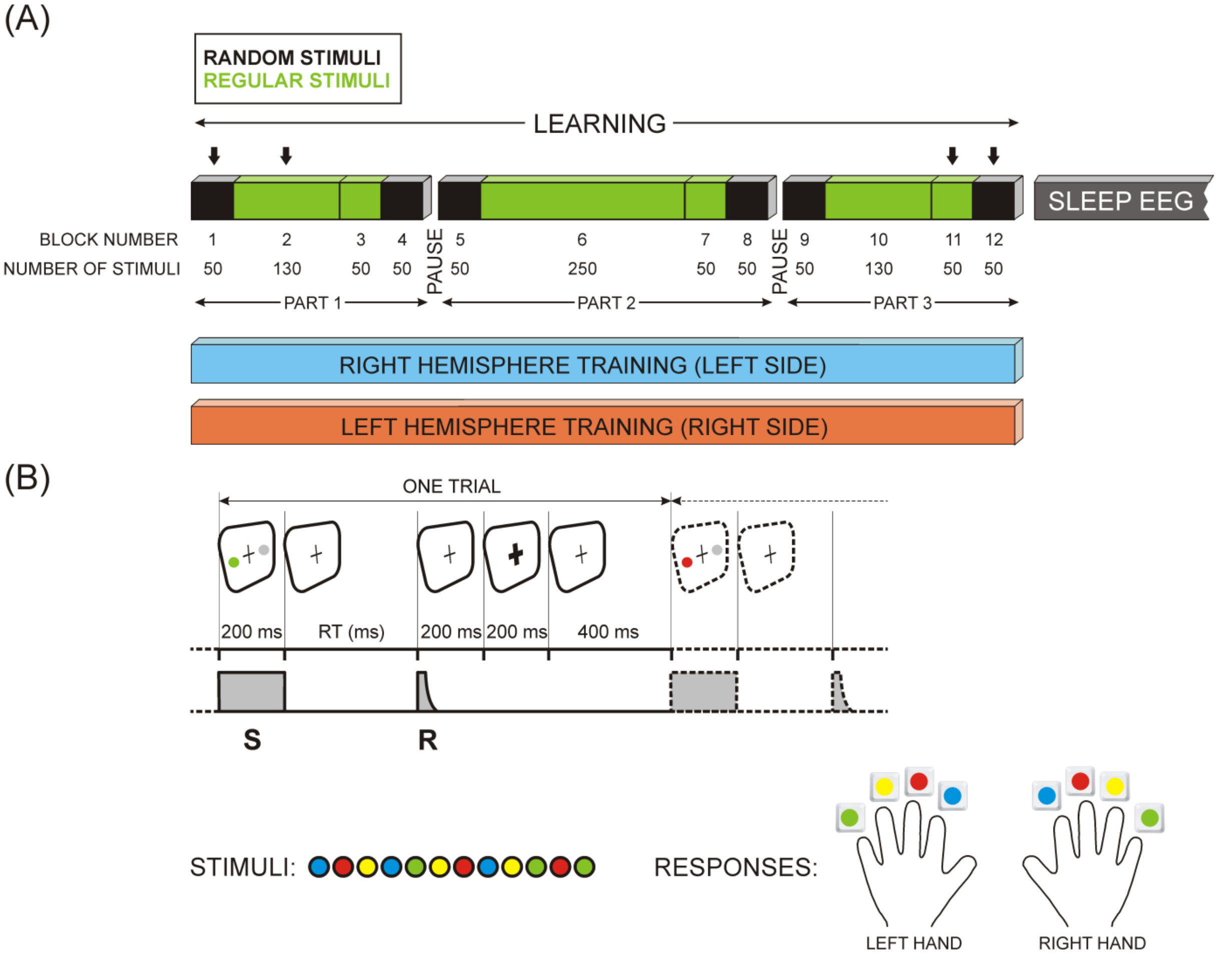

Supplementary Fig. S1. Graphic illustration of the experiment. **(A)** A serial response time task (SRTT) was performed in blocks numbered consecutively from 1 to 12 (**BLOCK NUMBER**), with the number of trials indicated below (**NUMBER OF STIMULI**). Different blocks contain random or regular stimuli as indicated in black and green, accordingly. After **PART 1** and **PART 2** a short break (**PAUSE**) was introduced. Detailed description of the task is presented in the text. Subjects practiced SRTT in an evening session (**LEARNING**), and performed an update session in the morning after sleep. Following the balanced design of the experiment, half of the participants learned the task either on the **LEFT** or on the **RIGHT SIDE** activating predominantly the contra-lateral right or left hemisphere (**RIGHT HEMISPHERE TRAINING, LEFT HEMISPHERE TRAINING**). Thick arrows denote the blocks used for analysis of EEG activity during learning. **(B)** Schematic presentation of one trial of the SRTT. Colour stimuli (**S**) last 200 ms and require button-press **RESPONSES** (**R**) with the respective finger as indicated in the lower panel. The order of repeated **STIMULI** in the regular blocks is presented also in the lower panel.

Stimuli

As shown in Fig. S1, each trial began with a fixation cross (black cross on a white screen). After 400 ms the cross was replaced by two circles of approx. 1 cm^2 each (with a diameter corresponding to 1° visual angle), one in colour and the other in grey, with equal displacement of 4.4^o visual angle from screen centre. In a given session, the colour circles appeared always right or always left, in one of the four colours green, blue, red, and yellow, always

counterbalanced by a grey circle at the opposite side. The two circles were presented for 200 ms and served as stimulus (S), after which the cross appeared again and lasted until a button was pressed (response R). If the response was correct, the cross changed after 200 ms for 200 ms to bold. Thereafter, the cross returned to its normal shape, and after 600 ms the next circles appeared. If the response was not correct, the cross did not change to bold and the next colour circle did not appear until the correct button was pressed.

Supplementary Results

Behavioural Results

The gain of implicit sequence knowledge (ImK) before sleep did not differ between the left- and right side groups, i.e., did not depend on the trained hemisphere. This was reflected by the lack of difference in the rate of participants from the two groups with statistically validated ImK gain (Yordanova et al., 2015). Results of the Chi-square statistics were: Right hemisphere trained, n = 8, Left hemisphere trained, n = 9 ($\chi^2(1/49) = 0.38$, $p = 0.54$). Also no significant effect of the Trained hemisphere on the ImK coefficient was found ($F(1/48) = 1.8$, $p = 0.2$). Nor did the Trained hemisphere affect the rate of full ExK generation after sleep (Right hemisphere trained n = 6, Left hemisphere trained, n = 5, $\chi^2(1/49) = 0.38$, $p = 0.54$).

Sleep Stages

Supplementary Table S1 presents the distribution of sleep stages in the non-learning and learning nights in left- and right-side learning groups. The distribution of the sleep stages, total sleep time and sleep efficiency did not differ between the groups in any of the nights ($F(1/47) < 3$, $p > 0.1$). Sleep efficiency in the two groups was higher in the learning as compared to the non-learning night ($F(1/47) = 68.6$, $p < 0.001$; Side (Trained hemisphere) x Night, $F(1/47) = 1.3$, $p > 0.2$).

Supplementary Table S1. Distribution of sleep stages in the non-learning and learning nights in left- and right-side learning groups.

Night	Non-Learning		Learning	
Learning<br>Trained hemisphere	Left-side<br>Right	Right-side<br>Left	Left-side<br>Right	Right-side<br>Left
W (%)	4 (4.1)	5 (3.6)	2 (5.3)	2 (2.4)
S1 (%)	5 (2.2)	5 (1.9)	2 (1.9)	2 (1.5)
S2 (%)	48 (7.3)	50 (5.1)	43 (6.7)	46 (6.4)
S3 (%)	13 (2.5)	13 (3.7)	16 (4.3)	16 (4.5)
S4 (%)	2 (1.9)	2 (1.5)	5 (3.3)	5 (3.0)
SWS (%)	15 (4.2)	15 (2.9)	21 (4.8)	21 (5.6)
REM (%)	26 (5.1)	24 (4.1)	31 (5.7)	28 (5.3)
TST (min)	465 (39)	466 (39)	453 (53)	466 (36)
SE (%)	94.6 (0.4)	95.3 (0.4)	96.6 (0.4)	97.6 (0.4)

Means and standard deviations (SD) are indicated. W: wake; S1: sleep stage 1; S2: sleep stage 2; S3: sleep stage 3; S4: sleep stage 4; SWS: slow wave sleep; REM: rapid eye movements (sleep); TST: total sleep time; SE: sleep efficiency = TST*100/(Total time in bed).

Topographic Distribution Analysis

Slow waves: As the topography maps in Fig. 2A show, the amplitude of the negative half wave of averaged slow oscillations (SWmin) was largest at frontal locations (Region, $F(7/45) = 311.5$, $p < 0.001$). The same results were yielded for the positive half wave of averaged slow oscillations (SWmax): Region $F(7/45) = 257.1$, $p < 0.001$).

SW-related slow spindle activity: As demonstrated by topography maps in Fig. 2B, slow spindle (9-12 Hz) activity, which was temporally coupled only with the down state of the SW (SWmin), was maximal at frontal locations (Region, $F(7/315) = 8.8$, $p < 0.001$), similar to SWA topography. Correspondingly, the coupling of slow spindle activity with SWmin also was only pronounced at frontal-central regions (Region $F(7/315) = 6.3$, $p = 0.01$).

SW-related fast spindle activity: As depicted in Fig. 2C, the magnitude of both SWmin- and SWmax-related fast spindle activity was maximally expressed at central locations (Region, $F(7/315) > 4.7$, $p < 0.001$). The coupling of SWmin and SWmax with fast spindle activity was maximal at frontal and parietal regions (Region, $F(7/315) > 2.4$, $p < 0.04$).
